# The Impact of Psychotropics on Sexuality: A Literature Review

**DOI:** 10.1192/j.eurpsy.2024.1600

**Published:** 2024-08-27

**Authors:** B. Abassi, F. Fekih-Romdhane, F. Baccar, M. Cheour, E. Sana, R. Damak

**Affiliations:** Ibn Omrane, Razi Hospital, Mannouba, Tunisia

## Abstract

**Introduction:**

Sexual dysfunctions related to psychotropic drugs are among the most distressing adverse effects and can lead to non-adherence to treatment.

**Objectives:**

To elucidate the mechanisms of psychotropic-induced sexual dysfunctions and to suggest strategies for their management.

**Methods:**

Literature review based on the keywords “psychotropics,” “sexuality,” and “sexual dysfunction”.

**Results:**

Psychotropic medications can impact sexuality either directly or indirectly. The direct effect is primarily due to a decrease in the mesocortical dopaminergic atmosphere, either by blocking D2 receptors or by stimulating 5HT2A receptors. D2 receptor blockade in the tubero-infundibular pathway triggers an increase in prolactin secretion, which can subsequently lead to erection problems, decreased libido, and difficulties achieving orgasm. Action in the nigrostriatal pathway may result in an extrapyramidal syndrome, which can, in turn, hinder intimate physical relations. The indirect effect can also be caused by metabolic complications, which are significant risk factors for sexual dysfunction, as they can lead to male hypogonadism and a negative self-image.

Therapeutic strategies suggest either reducing doses (if the patient’s condition allows), changing the drug, or adding an adjunctive medication. Aripiprazole, being a partial agonist of D2 and 5-HT1A receptors and an antagonist of D3 and 5-HT2A receptors, appears to cause fewer sexual dysfunctions and can reduce hyperprolactinemia when added to other antipsychotics.

**Conclusions:**

Healthcare professionals must proactively gather information on sexuality given its impact on quality of life and treatment adherence. Prioritizing this dimension of well-being demonstrates a respectful approach toward the patient and establishes the foundation for a strong therapeutic alliance.

**Disclosure of Interest:**

None Declared

